# What Topics Do Members of the Eating Disorder Online Community Discuss and Empathize with? An Application of Big Data Analytics

**DOI:** 10.3390/healthcare10050928

**Published:** 2022-05-18

**Authors:** Eunhye Park, Woo-Hyuk Kim, Sung-Bum Kim

**Affiliations:** 1 Department of Food Nutrition, College of BioNano Technology, Gachon University, Seongnam 13120, Korea; 2 Department of Consumer Science, College of Commerce and Public Affairs, Incheon National University, Incheon 22012, Korea; 3 Department of Business Administration, College of Business Administration, Inha University, Incheon 22212, Korea

**Keywords:** eating disorder, online community, Reddit, social support, topic modeling, big data

## Abstract

Given the stigma of eating disorders (EDs), anonymous online communities of individuals with EDs can play a critical role in their treatment. In our study, we aimed to identify prevalent topics related to EDs in one community. To discover latent topics in an online community dedicated to EDs, we applied an automated text-mining approach to topic modeling after collecting data from Reddit created between January 2011 and September 2020. As a result of topic modeling, topics regarding emotional support, informational support, and inquiries about EDs were discovered. In frequently asked questions and advice about EDs in the online community, community members empathized more with topics soliciting emotional support rather than informational support. Despite the importance of online communities, studies on this topic, especially those analyzing online conversations, have remained limited. By demonstrating the role of online communities in supplementary interventions, our findings can support clinicians in caring for patients with EDs.

## 1. Introduction

Eating disorders (EDs) involve bulimia and anorexia nervosa, binge-eating disorder, and specified feeding [1]. Eating disorders are a severe mental health concern, with important life-threatening medical consequences, psychiatric morbidity, reduced quality of life, and increased mortality [2]. Because the realities of EDs are often misunderstood and people with EDs are stigmatized as a result [3], recovery is jeopardized [4]. Beyond that, the shame and fear of stigma experienced by people with EDs can prevent them from seeking treatment in the first place, much less completing treatment successfully.

Social media platforms have increasingly become a popular space for individuals to share similar ideas, provide advice, seek information, discuss content, and assess themselves regarding their body and EDs through photos and videos [5]. Various topics involving physical and mental health topics are reviewed online among peers to produce vast amounts of content. The online ED community has gained attention in terms of two types of content (pro-ED and pro-recovery) categorized and posted on the social media platform [6]. Pro-ED content refers to “a desire to enact ED behaviors without indicating a desire to recover, while pro-recovery (or anti-ED) content is encouraging recovery from EDs and/or resistance to ED cognitions and behaviors” [7].

Many online pro-ED communities exist on social media platforms (e.g., Pinterest, Instagram, and Tumblr) [8], increasing their accessibility to vulnerable or impaired individuals. People at risk of EDs can selectively participate in various social media platforms. Passive engagement with image-based sites is negatively related with eating and body issues/concerns. Among individuals, including young and adolescent individuals, prior studies have reported a significant relationship between taking part in disordered eating behaviors and seeing online pro-ED content (e.g., [9]). To avoid public confrontation and prejudice, individuals with EDs or those who are curious about them tend to prefer anonymous online spaces, where they can freely discuss the topic with like-minded people without critical intrusion into their privacy [10]. In these spaces, they especially seek out health- or ED-associated information and/or social support from online communities on social media. Because such online communities and their anonymous forums facilitate open communication about EDs and offer opportunities for camaraderie while concealing users’ offline identities, individuals with EDs gravitate to them instead of seeking traditional treatment [11].

Interest in trends within social networks and the forms of social support that they can offer, especially regarding the impact of health conditions, has increased considerably. Social support is a meaningful element in the maintenance and development of EDs, and members of ED online communities tend to participate in community activities because they offer direct and indirect social support [11,12]. In particular, social media platforms provide important resources for exchanging social support in the context of recovering from EDs [10,13], typically informational support (e.g., facts, advice, and guidance) and emotional support (e.g., encouragement, sympathy, empathy, care, and comfort).

Against this background, we investigated the U.S.-based online platform, Reddit, to identify and analyze salient topics actively discussed in a representative ED online community, which influences the perceptions of its members. Reddit is a widely used social media platform for stigmatized illnesses [14]. Reddit, the world’s largest online community-based review site with thousands of communities in subreddits addressing specific topics, is a representative online platform that enables infomediation [15]. Since Reddit was launched in 2005 as an open, free source of information online, Reddit ranks among the largest, most visited English-language social media platforms. Specifically, approximately 18% of all Americans use it; and in 2021, it was classified as the tenth-most popular network of community in the United States [16]. Reddit supports anonymous, theme-specific forums called subreddits that allow exchanges between users as posters and commentators, all called “Redditors,” who engage in similar behaviors. On subreddits, responses to questions posed by users about interests, trends, patterns, experiences, and opinions can be viewed and voted on. Because Reddit is an online space for health-related information exchange, with content that provides a robust source of information [17], it can also function as a significant source of social support by allowing users to share their own experiences [18]. In turn, responses to questions or comments on ED-related issues raised in Reddit’s ED online community can offer a snapshot of the prevailing group-based advice and recommended interventions.

Some studies related to Reddit have been focused on and conducted recently [19]. Previous researchers have focused on the content of pro-ED content available on social media platforms and not on ED-related content [11]. Our study differed from studies reported in the literature, some of which have examined ED online communities and performed manual content analysis to characterize online text addressing EDs [7]. However, when time and other resources are limited, this approach restrains the amount of data that can be analyzed and can generate highly subjective results. Meanwhile, other studies have used network analysis to explore people’s perceptions of EDs as captured in text written on Twitter, the microblogging site [20,21]. In terms of text mining of Reddit as a representative of ED communities, the authors of [22] conducted text mining to evaluate and juxtapose interest using fitness tracking technology in terms of EDs in health-related online communities. In order to capture more insights into the role of online communities, our study generated empirical evidence of peoples’ perceptions of ED using text mining. More specifically, a topic modeling algorithm and network analysis were implemented to discover latent themes and topical structures from online discussions about ED. Based on the topical structures produced by the text-mining approach, researchers manually reviewed actual Reddit posts and comments to validate the topic modeling results and deliver more personal and detailed perspectives. Therefore, this study aimed to identify prevalent topics related to EDs in the online community. The specific research goals are (1) to discover the popularly discussed topics among ED community members in the anonymous online sphere, (2) to compare the differences in topical foci between original threads and comments in terms of topic popularity and content, and (3) to examine which topics were supported or disapproved by other members. Relevant studies report that to remain anonymous, most people (including sufferers) pursue and search online emotional social support or appropriate disease-associated information from online communities. However, for all online communities to provide healthy advice or recovery-oriented support is not possible [20]. This study provides guidelines for an anonymous social media platform with contemporary forums for individuals to seek help and guidance in terms of EDs.

## 2. Methods

### 2.1. Data Preparation

Reddit was selected for data collection. In our study, we were particularly interested in the subreddit concerning EDs (“r/EatingDisorders”), which, as of July 2021, had more than 55,000 members. In April 2021, we used Reddit’s Python-based application programming interface to collect all texts associated with threads (i.e., original posts) and comments in this subreddit, along with the creation dates and scores of each thread. As a result, we collected 11,484 documents generated between January 2011 and September 2020 (174 original threads and 11,311 comments associated with those threads).

Before conducting text mining, we performed text-cleaning procedures, including tokenization, lowercase conversion of all characters, stop word removal, lemmatization, and bigram creation. Once the documents were cleaned, we retained those with more than three words for topic modeling analysis, which amounted to a final dataset of 10,050 documents (167 original threads and 9883 comments). The number of original threads and comments for each time period is displayed in Table 1. In this dataset, the number of original threads and comments was greatest between 2019 and 2020, and each thread had approximately 60 comments on average.

### 2.2. Topic Modeling Analysis

To discover salient topics in Reddit’s ED online community, we followed an R-based probabilistic topic modeling approach called structural topic modeling (STM) [23]. Unlike the clustering approach, which classifies each document into the single-most salient category, STM calculates the probabilities of each document in relation to multiple topics. Because a document often discusses multiple topics, probabilistic topic modeling is appropriate for processing our dataset. In this approach, documents with a strong focus on a particular subject are likely to have an exceptionally high probability (i.e., close to 1) of being classified into one topic and very low probabilities of being classified into other topics (i.e., close to 0). By contrast, if documents address multiple subjects, then they are likely to have moderately high probabilities of being classified into multiple topics.

**Figure 1:**
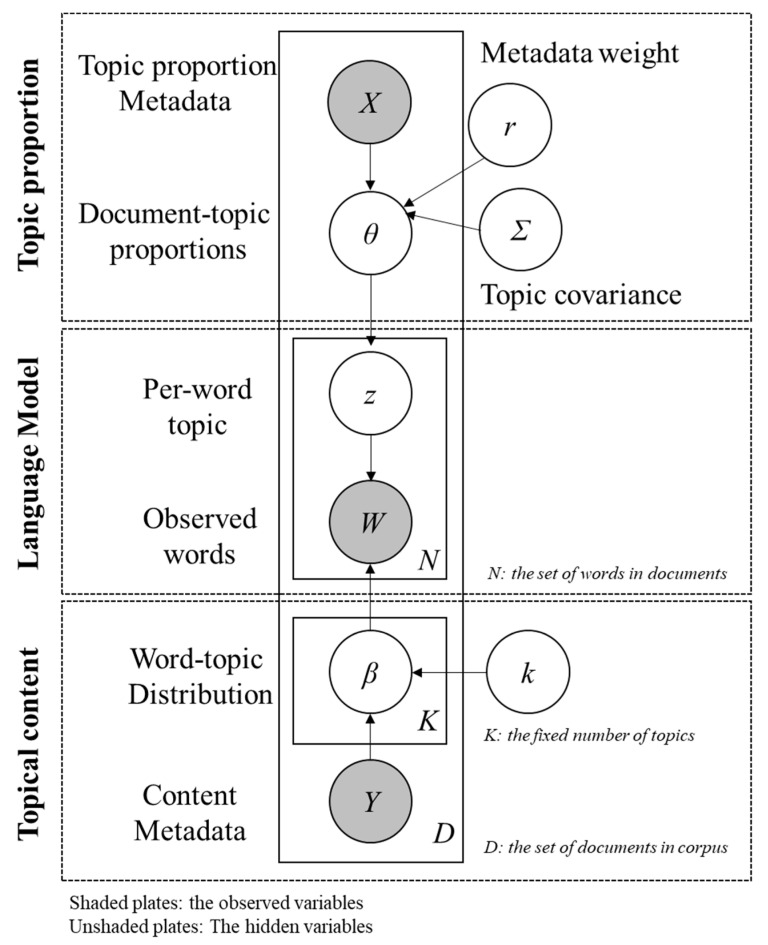
Graphical illustration of STM (adapted from Roberts et al. [23]).

Topic modeling algorithms require investigators to input the optimal topic number (K) for the dataset. Thus, the quantitative index (i.e., held-out likelihood and semantic coherence scores) was compared to evaluate the topic modeling performance for the models with consecutive topic numbers, from 3 to 50. Based on the quantitative index results and topic modeling results, researchers manually reviewed the topic modeling results between 10 and 15 topics, and agreed that 14 topics were appropriate for our dataset. Over the course of multiple iterations, STM automatically built the model and generated a list of words that constitute each topic (β) and the probability of each topic being associated with each document (θ), as shown in Figure 1. Because STM follows a probabilistic approach, the results can differ each time the model is run. Therefore, to ensure the reproducibility of the results, we set a seed in the model. Moreover, to comprehend the meanings of the topics and label them, we manually reviewed the top words of the topics and the documents highly associated with each topic, which can be discerned by arranging topic proportion scores per document in descending order. In summary, this study implemented mixed methods of machine-learning-based text mining and a traditional qualitative approach to produce semantically coherent and comprehensible results.

Of all topic modeling algorithms, STM has been widely applied because of its strength in covariate estimation [24,25,26]. As shown in the graphical visualization of STM (see Figure 1), STM enables the inclusion of both topic proportion metadata (X) and topic content metadata (Y) to estimate the effects of metadata. We selected the type of Reddit post (i.e., original thread vs. comment) as document-level metadata to examine the effects on topic proportion and topic content. Because the members of any online community may create new threads to share their experiences or ask questions, and other members leave comments to sympathize and/or share their opinions or advice regarding the issue raised, the contents of threads demonstrate the issues that concern many community members. The contents of comments showcase advice and/or solutions from people who may have experienced similar issues. To discover potential differences in intentions and sentiments between the ED community members who make original threads and leave comments, we compared topic proportion (θ) and topic content (β) between the original threads and comments. The results of comparing topic proportions indicated the types of Reddit documents (i.e., original threads vs. comments) mentioned a certain topic more frequently, whereas the result of comparing topic content revealed whether the same topic was expressed differently using distinct terms.

### 2.3. Topic Network Analysis

Using the results of topic modeling, we performed a topic network analysis in order to determine the hierarchical structure of the 14 extracted topics. STM offers a function of “topicCorr,” which enables the estimation of correlations with the document-topic proportion distributions. The topic network was generated through topic correlation estimations, and community detection was applied to the topic network. In topic network analysis, topics that share characteristics can be grouped to create communities, which we accomplished with igraph package from R software. Following Girvan and Newman [27], who proposed the modularity score as a mathematical framework for evaluating community structure in networks, we compared modularity scores across various community detection algorithms to select the appropriate method. Modularity is estimated by comparing the sum of connections for all edges to nodes in the community with the sum of connections to nodes at random. Hence, high modularity represents a densely structured community that connects many internal nodes and few external nodes [27]. To discover communities that consist of internally coherent and tightly connected topics, we selected the “walktrap” community detection algorithm [28], which had the highest modularity score.

### 2.4. Estimation of Topic Structure on Post Score

Members of a subreddit can “upvote” or “downvote” a thread to express their approval or disapproval of the thread, and the score of each thread represents the net score of votes (i.e., the number of upvotes minus downvotes). Thus, the threads that resonate with more users are likely to score higher, whereas threads that address controversial issues are likely to score lower. It is assumed that the net score of votes for each thread represents how much other users agreed with not only the original thread, but also the discussions in the comments. Hence, all documents belonging to each thread were assigned the same net score as the original thread. To identify which threads tended to have higher scores, we performed multiple regression analysis between topic proportions (θ) and scores in SPSS 21.0. In that analysis, we used document-level topic proportion as an independent variable and the net score of votes as a dependent variable. Because the sum of topic proportions per document is always 1, using raw topic proportion scores can cause serious multicollinearity in the regression model. To counter this risk, we recorded the topic proportion scores. Antons et al. [29] proposed that if the topic proportion exceeded 0.1, then the document addressed the corresponding topic. Following this logic, we converted topic proportions exceeding 0.1 to 1 and all others to 0.

## 3. Results

### 3.1. Topic Structure Discovered in the ED Online Community

STM estimates the associations (i.e., β: word-topic distribution) between 14 topics and vocabulary in the corpus. The top five words that are most closely associated with each topic with the highest β are listed in Table 2. STM identified the 14 most salient topics from discussions in the online community; of them, the three with the highest topic proportions were communication (Topic 8), binge eating (Topic 13), and professional help (Topic 10). Figure 1 illustrates the top words that appeared frequently in those three topics, with larger words indicating a higher frequency in the dataset.

The most popular topic, communication (Topic 8), accounted for 13.1% of the total topic proportion; the top words associated with the topic were “talk,” “tell,” “want,” “friend,” and “support,” thereby indicating that the importance of communication as social support in the ED online community. The topic consisted of discussions about issues related to communicating about EDs. More specifically, members actively shared their opinions about whether seeking help by communicating their own or close ones’ ED symptoms with others was necessary and about the issues that they need to consider when communicating those symptoms.

Another popular topic in the ED online community was binge eating (Topic 13), which accounted for 10.5% of the total topic proportion. The top words associated with Topic 13 were “eat,” “food,” “binge,” “body,” and “feel,’ which indicates members’ concerns about binge eating and its impact on their body’s conditions and emotions. The documents most closely associated with binge eating offered practical tips and advice to overcome the ED and to establish healthy eating routines. The topic of professional help (Topic 10), with the top words “help,” “therapist,” “need,” “doctor,” and “therapy,” accounted for 9.5% of the total topic proportion. The documents associated with the topic mostly concerned the importance of finding professional help to overcome ED. In particular, many community members advised others to access specialists in EDs.

### 3.2. Topic Network

Using the results of topic modeling, we implemented a fast greedy algorithm that automatically detected four communities that maximized the modularity scores. In Community 1, we grouped two topics related to emotional support in order to boost self-esteem, namely self-esteem for existence (Topic 1) and self-esteem for health (Topic 5). In Community 2, we grouped five topics related to informational support, meaning a question about or recommendations for managing ED-related issues, including ED-related health issues (Topic 7), communication about EDs with others (Topic 8), and professional help (Topic 10). Another topic in Community 2 represented the fact that individuals sometimes seek cooperation from the ED online community for their research (Topic 2). Next, the topics belonging to Community 3 were feeling in control (Topic 3), recovering from EDs (Topic 4), snacking behavior (Topic 6), binge eating (Topic 13), and exercise routine (Topic 14). Community 2 and Community 3 are both related to social support for ED community members. However, whereas the topics in Community 2 were more applicable to individuals at the initial stage of admitting ED symptoms, those in Community 3 were more applicable to individuals already in the process of overcoming EDs. Last, Community 4 concerned inquiries about ED in topics related to EDs (Topic 9) and ED diagnosis (Topic 12). Topic 9 also included factors or aspects of personal background that may be associated with the occurrence of EDs or challenges in overcoming EDs. On that topic, many community members shared research findings or their own experiences, as illustrated in the following three documents:

“(…) My ED tendencies started immediately following having jaw surgery, && having my jaw wired shut for a few weeks. (…)”

[Comment on a post written in March 2019]

“(…) I studied the link between eating disorders ED and autoimmune diseases AD, which is growing more and more evident to the point that many doctors will screen for an ED after a AD diagnosis. (…)”

[Comment on a post written in March 2019]

“(…) Another man with an eating disorder here. For your reference, I am in a support group for men with EDs, and there are 50/50 straight/gay men in it. (…)”

[Comment to a post written in April 2014]

### 3.3. Topic Proportion Comparisons between Original Threads and Comments

Two topics were more prevalent among the original threads than comments: research requests and resource seeking (Topic 2) and recovering from EDs (Topic 4). Because researchers have used the online community to request members’ participation in their research, the topic proportion of Topic 2 was relatively high among the original threads. The same dynamic occurred in Topic 4 because many people requested other members’ advice on recovering from EDs.

Three topics were mentioned more frequently in comments than in original threads: self-esteem for existence (Topic 1), various issues related to ED (Topic 9), and feeling in control (Topic 3), thereby demonstrating the most popular comments or recommendations in response to those ED-related questions. As shown in various topics offering practical information to overcome EDs, providing useful information plays a critical role in the online community. Additionally, comments about having control over self-esteem and feelings were also prevalent. The popularity of topics regarding various issues related to ED indicates that many members of the ED community have acknowledged that EDs have complex symptomology involving various situations or factors that trigger ED.

### 3.4. Topic Content Comparisons between Original Threads and Comments

**Figure 2:**
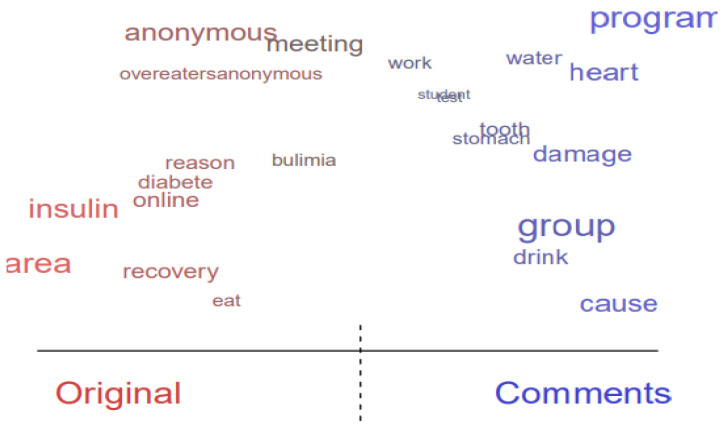
Difference in topic content between original threads and comments (health issue: Topic 7).

Topic content was compared to identify potential gaps between the original threads and comments. Words skewed to the left side (i.e., “Original”) were more frequently used in original posts, whereas words skewed to the right side (i.e., “Comments”) were more frequently used in comments. The words placed in the middle were commonly used in both original posts and comments, and the word size represents the frequency of the terms in the dataset. Regarding ED-related health issues (Topic 7), original threads offer brief explanations about health conditions (e.g., diabetes) that trigger EDs or physiological complications due to the disorders (Figure 2). Some community members asked questions about joining support groups (e.g., Overeaters Anonymous) to recover from health issues associated with EDs. In the comments, many users shared detailed explanations about bodily damage or diseases that EDs can cause, including heart damage, kidney failure, problems with fluid balance, and tooth decay.

**Figure 3:**
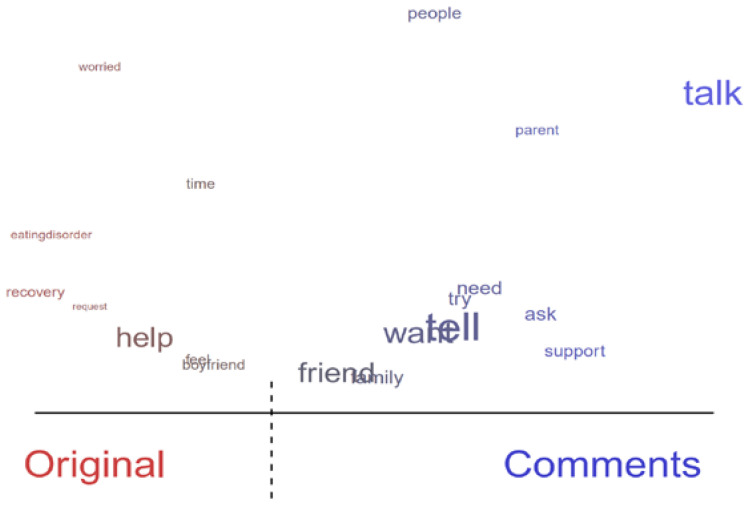
Difference in topic content between original threads and comments (communication: Topic 8).

For the topic of communication (Topic 8), the term “boyfriend” was frequently used in original threads, whereas “family” and “friends” were frequently used in comments (Figure 3). The original posts related to Topic 8 indicate that for individuals in a significant relationship, their partners are usually the ones who notice EDs in the early stage, because they share large portions of their daily lives together as well as intimacy. As such, partners often play a significant role in the uncovering of ED symptoms. In the comments, the roles of family and friends, however, were more often emphasized as sources of social support, as discussed in Section 3.1. In any case, because sensitive issues may be discussed in communicating about EDs, such communication requires an informed understanding of EDs from the family and friends of people with EDs.

**Figure 4:**
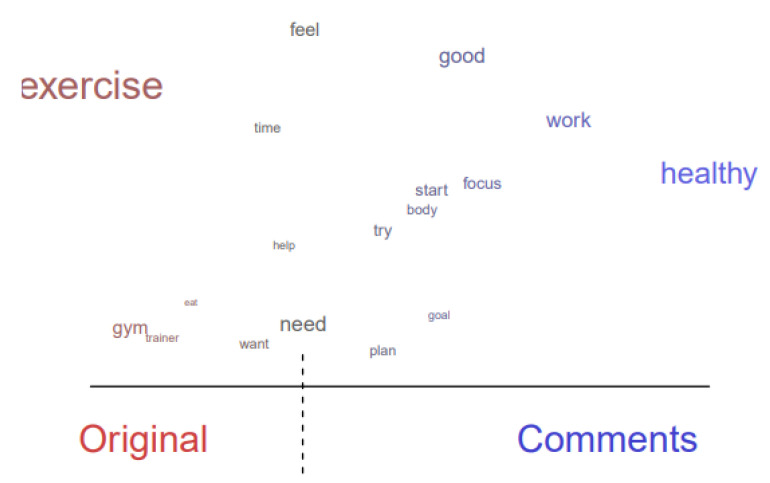
Difference in topic content between original threads and comments (exercise routine: Topic 14).

Among original threads related to the topic of exercise routine (Topic 14), the terms “want,” “need,” and “help” appeared frequently, thereby indicating that community members have acknowledged the importance of exercise in overcoming EDs (Figure 4). Even so, the terms indicating the actual performance of exercise were absent. In the comments, the term “healthy” appeared most frequently and is shown to be closely related to comments by being skewed to the right side. This trend indicates that users who left comments commonly mentioned that the goal of exercise should be health, not other reasons. Moreover, the frequent appearance of the terms “start,” “try,” “plan,” and “goal” indicate that many users commented on the importance of taking action, setting clear goals, and recognizing achievements.

### 3.5. The Effects of Topical Structure on the Net Score

To identify which topics were largely supported by members of the online ED community, we performed regression analysis to gauge the effects of topical structures on net scores (Table 3). That is, whether certain topics had higher or lower votes from other ED members. In addition to the regression coefficient, topic weights were considered to classify the topics into two categories: topic popularity and votes. Topic popularity was determined based on topic weights. If a topic had a topic weight greater than the median topic weight, this topic was classified as high popularity, while a topic with lower topic weight was classified as low popularity.

Three topics had significantly positive regression coefficients: self-esteem for existence (Topic 1, *p* < 0.001), feeling in control (Topic 3, *p* < 0.05), and self-esteem for health (Topic 5, *p* < 0.001), suggesting that many members of the ED community supported the underlying ideas of those topics. In general, all these topics are related to emotional support. In contrast, five topics had a negative significant regression: research requests and resource seeking (Topic 2, *p* < 0.001), snacking behavior (Topic 6, *p* < 0.05), various issues related to EDs (Topic 9, *p* < 0.05), professional help (Topic 10, *p* < 0.001), and ED diagnosis (Topic 12, *p* < 0.001). These topics, all related to informational support, tended to have negative coefficients. Finally, four topics had insignificant coefficients: ED recovery (Topic 4, *p* > 0.05), communication (Topic 8, *p* > 0.05), binge eating (Topic 13, *p* > 0.05), and exercise routine (Topic 14, *p* > 0.05). Except for exercise routine (Topic 4), all three topics had topic weights greater than the median topic weights, indicating that many ED members were interested in these topics. Hence, for these three topics, it is plausible that those topics had insignificant coefficients because they are controversial. That is, they have high upvotes and downvotes, which reduced their net scores. However, the exercise routine topic (Topic 4) had a low topic weight, indicating that the discussion about this topic was not active among the community members. Therefore, it is possible that members of the ED online community are not interested enough in Topic 4 to vote on them.

## 4. Discussion

Although social media platforms have inadvertently offered a space for the proliferation of pro-ED content (e.g., [30]), they simultaneously offer rooms for pro-recovery communities, where people with EDs are able to share their own experiences and offer care to one another (e.g., [31]). Moreover, there have been few empirical studies focusing on ED content from online communities (i.e., Reddit). This study captures the topics of online communication about EDs that have been prevalent in an ED online community [32]. By doing so, we discovered the most prevalent ED-related questions and associated responses from online community members. The findings can be valuable for both patients with EDs and clinicians by presenting the usefulness of such online communities for free, open, anonymous discussions about EDs without confronting stigma.

Previous studies have focused on the nature and content of pro-ED communities that promote EDs as a lifestyle on different social media using content or network analyses [12,32]. Relevant research has also investigated the association between the prevalence of EDs and their related factors [1]. Most ED research employs self-report measures [33]. Reddit is the world’s largest online community-based review site as a significant source of information. Nevertheless, there are limited studies on how people seek and share the physical and mental health-related information and content on the platform [34]. Therefore, research addressing how Redditors respond to health-related information using big data approaches is scarce. In that light, our study makes contributions to ways of investigating ED-related research questions using big data approaches.

This study is grounded on “social support and computer-mediated communication theory”, which indicates online media can serve as a tool to help individuals with physical and mental health [35]. Based on such assumption, this study achieved the research goals of this study. The first research objective was to uncover most popularly discussed topics at the online community. By implementing a text-mining approach to discover the most popularly discussed topics in the ED community, we discovered 14 salient topics that were commonly shared by members, which represents the commonly perceived issues by ED-community members. Out of 14 most frequently discussed topics, people frequently talked about the importance of getting professional help, how to improve self-esteem and how to recover from EDs.

We also analyzed the topical contents of both original threads and comments. Original threads are mostly questions regarding EDs written by those who are suffering from the symptoms while comments tend to be advice for the corresponding questions written by those who are going through or overcame EDs. By comparing original threads and comments, we discovered the most frequently questioned concerns and types of advice. EDs are mental illnesses at the base, advice related to self-esteem, feelings, and subjective self-evaluations had great weight and value in the ED online community, and the relevant topics were popularly mentioned and advised.

Finally, we were able to identify which topics were approved or disapproved by other members by using the vote score on each post. Voting behaviors can be considered as an intervention or an indirect participation in the communication to solve the issues and support the members with the problems. Such an approach is useful to capture the perceptions of members who actively participate in the online community by creating posts and leaving comments, as well as inactive members who browse posts, comments, and votes.

Popularity and voting patterns were used as criteria to classify the topics into the following categories: popular and upvoted, popular and downvoted, unpopular and upvoted, unpopular and downvoted, popular and controversial, and unpopular and uninterested. These findings demonstrate ED-related topics that many people are interested in and group-based opinions toward topics shaped by online community members. For instance, the topic related to self-esteem in relation to existence was shared in comments and received many upvotes, indicating that many ED members agreed on this idea and were willing to share their opinions through active participation in commenting to original threads. On the other hand, the topic regarding professional help was frequently mentioned in the ED online community, but this topic tended to be downvoted. Despite the popularity of this topic, ED members disapproved posts or comments about the topic regarding professional help because they believe it is not appropriate to seek professional help in the ED community, which mostly consists of non-experts. In other words, ED members agreed that questions about professional help should be discussed with health care professionals or experts rather than the ED community.

The current study revealed the role of ED online communities by examining how ED online community members communicate with each other to deal with EDs. Many community members sympathized more with topics soliciting emotional support rather than those soliciting informational support. Therefore, the role of ED online communities should be emphasized as a means of gaining emotional support instead of searching for factual information. Moreover, people who use ED online communities should carefully consider that most community members are not professionals but merely people who are interested in EDs or who have or have had EDs. It is plausible, therefore, that some posts in the community may contain incorrect information, which demands caution from members when reviewing the information.

## 5. Conclusions

Our findings can help individuals with symptoms of ED and clinicians who treat them. For one, we found that many people freely share their symptoms and situations related to EDs in online spaces, where anonymity is guaranteed. Because online communities are regarded as protected from the judgment of others, they are open to anyone with concerns about EDs, including pre-eating disorders. Thus, online communities can be a medium to inform patients and facilitate changes in their thoughts and behaviors before their symptoms progress to a serious degree.

Another benefit of the online communities is that they are easily available to those suffering from EDs as well as those who are close to the patients. We discovered that friends and family members of people with ED symptoms commonly used online communities to seek for advice. As a result, clinicians can utilize online platforms to understand difficulties affecting ED patients and those close to them. Despite the benefits of the online communities outlined, most online community contents are emotional support from non-experts rather than informational support from professionals. Hence, in the future, online service through readily accessible media will be required so that people suffering from EDs can receive credible information and guidance from experts without too much difficulties.

Based on the limitations of the current study, we propose the following tasks for future research. The current study is limited to ED communities on Reddit with a limited number of original threads, so the results cannot be generalized to other communities. Therefore, future study may target different types of online discussion at different social media platforms, such as Twitter and Facebook. Since this study analyzed Reddit posts generated over several years, people’s perceptions may have changed, which requires future studies to compare the differences in people’s perceptions and opinions. For the regression analysis, this study assigned the same net scores of the original threads to the comments based on the assumption that other users vote the thread after reading both the original threads and comments. However, some people may disregard the comments and vote on the threads solely based on the original thread content. Future studies are necessary to overcome such limitations.

## Figures and Tables

**Table 1:** Summary of final dataset.

Time Period	Original Threads	Comments	Total Documents
2011–2012	12	341	353
2013–2014	28	1143	1171
2015–2016	19	642	661
2017–2018	44	1288	1332
2019–2020	64	6469	6533
Total	167	9883	10,050

**Table 2:** Summary of topics in the ED online community.

Category	Topic Number	Label	Topic Proportion	Top Words
Category1: Emotional support for self-esteem	Topic 1	Self-esteem: existence	0.087	love, recovery, people, life, want
	Topic 5	Self-esteem: health	0.056	look, feel, body, healthy, sound
Category 2: Informational support and assistance: Early stage	Topic 2	Research request and resource seeking	0.051	book, research, free, resource, link
	Topic 7	Health issues	0.035	cause, program, stomach, damage, group
	Topic 8	Communication	0.131	talk, tell, want, friend, support
	Topic 10	Professional help	0.095	help, therapist, need, doctor, therapy
	Topic 11	Gratitude to support	0.081	thank, hope, post, advice, comment
Category 3: Informational support and assistance: In progress	Topic 3	Feeling control	0.045	feel, try, walk, watch, feeling
	Topic 4	ED recovery	0.086	year, time, start, recovery, weight
	Topic 6	Snacking behavior	0.064	food, eat, meal, try, snack
	Topic 13	Binge eating	0.105	eat, food, binge, body, feel
	Topic 14	Exercise routine	0.038	exercise, work, time, run, healthy
Category 4: Inquiries about ED	Topic 9	Various issues related to ED	0.043	diet, fat, body, woman, addiction
	Topic 12	ED diagnosis	0.084	weight, people, disorder, anorexia, behavior

**Table 3:** Regression result.

Category	Model		Topic Weights	Beta	Std. Error	t	Sig.	Classification	
								Popularity	Vote
Category 1: Emotional support for self-esteem	T1	Self-esteem: existence	0.087	3.386	0.967	3.502	***	High	Upvoted
	T5	Self-esteem: health	0.056	8.324	1.229	6.773	***	Low	Upvoted
Category 2: Informational support and assistance: Early stage	T2	Research request and seeking	0.051	−8.871	1.302	−6.811	***	Low	Downvoted
	T7	Health issues	0.035	−8.430	1.526	−5.526	***	High	Downvoted
	T8	Communication	0.131	0.702	0.880	0.798		High	Controversial
	T10	Professional help	0.095	−8.829	0.950	−9.288	***	High	Downvoted
	T11	Gratitude to support	0.081	2.553	1.027	2.486	*	High	Upvoted
Category 3: Informational support and assistance: In progress	T3	Feeling control	0.045	3.246	1.379	2.355	*	Low	Upvoted
	T4	ED recovery	0.086	−1.104	0.954	−1.157		High	Controversial
	T6	Snacking behavior	0.064	−2.618	1.218	−2.150	*	Low	Downvoted
	T13	Binge eating	0.105	−1.036	0.975	−1.062		High	Controversial
	T14	Exercise routine	0.038	−1.631	1.672	−0.975		Low	Uninterested
Category 4: Inquiries about ED	T9	Various issues related to ED	0.043	−2.885	1.393	−2.071	*	Low	Downvoted
	T12	ED diagnosis	0.084	−5.032	1.005	−5.005	***	High	Downvoted
	Constant			40.777	1.131	36.053	***		

DV: score, * *p* < 0.05, *** *p* < 0.001.

## Data Availability

Not applicable.
